# Treatment Response to Botulinum Neurotoxin-A in Children With Cerebral Palsy Categorized by the Type of Stretch Reflex Muscle Activation

**DOI:** 10.3389/fneur.2020.00378

**Published:** 2020-06-02

**Authors:** Lynn Bar-On, Erwin Aertbeliën, Anja Van Campenhout, Guy Molenaers, Kaat Desloovere

**Affiliations:** ^1^Department of Rehabilitation Medicine, Amsterdam UMC, Amsterdam Movement Sciences, Amsterdam, Netherlands; ^2^Department of Rehabilitation Sciences, KU Leuven, Leuven, Belgium; ^3^Department of Mechanical Engineering, KU Leuven, Leuven, Belgium; ^4^ROB Core Lab, Flanders Make, Leuven, Belgium; ^5^Department of Development and Regeneration, KU Leuven, Leuven, Belgium; ^6^Clinical Motion Analysis Laboratory, University Hospital Leuven, Leuven, Belgium

**Keywords:** cerebral palsy, stretch reflex, spasticity, gait analysis, Botulinum toxin, treatment, tone reduction, muscle spindles

## Abstract

While Botulinum NeuroToxin-A (BoNT-A) injections are frequently used to reduce the effects of hyperactive stretch reflexes in children with cerebral palsy (CP), the effects of this treatment vary strongly. Previous research, combining electromyography (EMG) with motion analysis, defined different patterns of stretch reflex muscle activation in muscles, those that reacted more to a change in velocity (velocity dependent –VD), and those that reacted more to a change in length (length dependent –LD). The aim of this study was to investigate the relation between the types of stretch reflex muscle activation in the semitendinosus with post-BoNT-A outcome as assessed passively and with 3D gait analysis in children with spastic CP. Eighteen children with spastic CP (10 bilaterally involved) between the ages of 12 and 18 years were assessed before and on average, 8 weeks post-treatment. EMG and motion analysis were used to assess the degree and type of muscle activation dependency in the semitendinosus during passive knee extensions performed at different joint angular velocities. Three-dimensional gait analysis was used to assess knee gait kinematics as a measure of functional outcome. Pre-treatment, 9 muscles were classified as VD and 9 as LD, but no differences between the groups were evident in the baseline knee gait kinematics. Post-treatment, stretch reflex muscle activation decreased significantly in both groups but the reduction was more pronounced in those muscles classified pre-treatment as VD (−72% vs. −50%, *p* = 0.005). In the VD group, these changes were accompanied by greater knee extension at initial contact and during the swing phase of gait. In the LD group, there was significantly increased post-treatment knee hyperextension in late stance. Although results vary between patients, the reduction of stretch reflex muscle activation in the semitendinosus generally translated to an improved functional outcome, as assessed with 3D gait analysis. However, results were less positive for those muscles with pre-treatment length-dependent type of stretch reflex muscle activation. The study demonstrates the relevance of categorizing the type of stretch reflex muscle activation as a possible predictor of treatment response.

## Introduction

Cerebral palsy (CP) is a common childhood physical disability caused by a non-progressive brain injury resulting in impaired development of the musculoskeletal system (1). The most common motor impairment among children with CP is spastic paresis (2), in which hindered development of the descending motor and sensory tracks results initially, in muscle paresis, followed very quickly by muscle alterations including increased sensitivity of the stretch reflexes (3, 4). When stretch reflexes, as measured at rest, are exaggerated in response to a changing stretch velocity, they are referred to as “spasticity” (5). In addition, studies carried out on different muscles of children with CP have reported exaggerated stretch reflexes in response to changes in muscle length, rather than velocity (6–9). In particular, length-dependent stretch reflex activation was most prevalent in the semitendinosus (6). Some hypothesize that this length-dependency reflects more severely affected muscles (2) or muscles with a more complex movement disorder (10). While several underlying pathophysiological mechanisms of the different types of stretch reflex muscle activation have been proposed (2), it is also relevant to verify whether treatment response differs between the two types of stretch reflex activation.

Reduction of hyperactive stretch reflexes is a key component of the therapy directed at children with spastic CP. For example, injections of Botulinum NeuroToxin-A (BoNT-A) to the semitendinosus are commonly included in a multi-level therapy approach (11). However, treatment success following BoNT-A injections varies strongly, especially in proximal leg muscles, such as the semitendinosus (12). This response variability necessitates more research to help delineate to whom such treatment should be directed. This is especially important given the concerns regarding the possible longitudinal impact of repeated BoNT-A injections on muscle growth (13, 14).

Instrumented assessments such as electromyography (EMG) and 3D motion analysis are becoming more accessible in clinical settings. Compared to ordinal clinical scales, these instruments allow more accurate diagnosis of impairments in children with CP, leading to more informed treatment decisions. In addition, given the low correlation between impairments assessed with the child at rest on an examination table and their assessment during gross motor function, such as gait, it is important that treatment effects are assessed in an instrumented way in both passive and active conditions (15). Therefore, using EMG and motion analysis during passive stretch and during gait, the aim of this study was to examine whether the type of stretch reflex muscle activation affects treatment outcome after BoNT-A injections in the semitendinosus of children with spastic CP. We hypothesized that those muscles with pure velocity-dependent reflexes pre-treatment will show greater improvement post BoNT-A compared to the muscles that show clear length-dependent reflex activations.

## Methods

### Ethics Statement

Ethical approval was granted by the University Hospitals' Ethics Committee (B32220072814). Parents/guardians and subjects were informed of the procedure. Parents/guardians and children over the age of 12 provided written informed consent in accordance with the Declaration of Helsinki.

### Participants

A convenience sample of 19 children with spastic CP aged 12–18 years were included (Table 1). Data from the instrumented spasticity assessment, but not their gait analysis, had also been analyzed for a previous study (16). One child from this sample did not undergo a gait analysis on the same day as the instrumented spasticity assessment and was therefore excluded from the current study, resulting in 18 subjects for final analysis. The exclusion criteria were as previously reported: a diagnosis (by a neurologist) of ataxia or dystonia; severe muscle weakness [<2+ on the Manual Muscle Test (17)]; poor selectivity (18); knee joint contractures that compromised passive knee joint motion to <20 degrees; cognitive problems that could impede the measurements; previous lower-limb orthopedic surgery; intrathecal baclofen pump; selective dorsal rhizotomy; BoNT-A injections 6 months prior to the first assessment.

**Table 1:** Included subject characteristics.

		**Pre-treatment stretch reflex activation**	
	**All (*n* = 18)**	**Velocity dependent (*n* = 9)**	**Length dependent (*n* = 9)**
Mean age (SD) (years)	9.78 (1.87)	9.65 (1.77)	9.89 (2.07)
Male/female (n)	12/6	6/3	6/3
Bilateral involvement (n)	11	4	7
Unilateral involvement (R/L) (n)	3/4	3/2	1/1
GMFCS (I/II/III) (n)	6/8/4	5/3/1	1/5/3
Median (range) number of previous BoNT-A injections in the medial hamstrings (semitendinosus, semimembranosus, gracilis)	2 (0–9)	2 (0–9)	0 (0–5)

*GMFCS, gross motor functional classification scale; BoNT-A, botulinum neurotoxin-A*.

### Treatment

Subjects received BoNT-A injections in the semitendinosus as part of a multilevel treatment. BoNT-A dosage and muscle selection were based on patient weight, medical history, findings of a clinical examination, and 3D gait analysis. Injection was done under short general anesthesia, and ultrasound was used for visual identification of muscles and needle depth control (19). As part of the established and standardized integrated approach for BoNT-A treatment at the University Hospital, all patients received some casting during 1–2 weeks immediately following the injections. Some children only had ankle casting (i.e., below-the-knee), while for other children the ankle casts were combined with removable or non-removable full leg casts and an additional abduction-exorotation bar. As part of their regular care, physiotherapy was intensified during the first 4 weeks post-treatment such that three to five sessions of 30–60 min physiotherapy were provided per week. Details of the treatments are reported in Table 2.

**Table 2:** Treatment details: number of injected muscles, average amount (SD) of Botulinum neurotoxin-A (botox®) injected per muscle, number of subjects prescribed with upper leg casts and number of lower and/or upper-leg casts, average number of casting days, physiotherapy sessions per week and duration.

			**Pre-treatment stretch reflex activation pattern**			
	**All (*****n*** **=** **18)**		**Velocity dependent (*****n*** **=** **9)**		**Length dependent (*****n*** **=** **9)**	
botox®	*n*	Units/kg	*n*	Units/kg	*n*	Units/kg
Psoas	13	1.85 (0.55)	6	1.50 (0.55)	7	2.14 (0.38)
Adductor	7	1.21 (0.57)	3	0.83 (0.29)	4	1.5 (0.58)
Rectus femoris	3	1.0 (0.50)	2	1.00 (0.71)	1	1.0 (0.0)
Medial hamstrings (semitendinosus, semimembranosus, gracilis)	18	4.44 (1.09)	9	4.32 (0.97)	9	4.56 (1.24)
Gastrocnemius	13	3.04 (1.33)	7	2.93 (1.64)	6	3.17 (0.98)
Soleus	8	1.91 (0.63)	4	1.69 (0.63)	4	2.13 (0.63)
Casting	*n*	Days in casts	*n*	Days in casts	*n*	Days in casts
Removable upper-leg casts	13	12.67 (1.97)	7	12.50 (2.07)	6	13.00 (2.00)
Non-removable upper-leg casts	4		2		2	
No upper-leg casts	1		0		1	
Non-removable lower-leg casts	16		9		7	
Physiotherapy	Sessions/week	Session duration (min)	Sessions/week	Session duration (min)	Sessions/week	Session duration (min)
	4.72 (2.37)	36.94 (12.14)	4.44 (1.01)	37.22 (11.74)	5.00 (3.28)	36.67 (13.23)

### Data Collection and Processing

#### 3D Gait Analysis

Three-dimensional gait analysis data were collected using a 12 camera VICON system, operating at 100 Hz (VICON, Oxford Metrics, Oxford, UK). Fifteen reflective markers were placed at specific anatomical landmarks on the pelvis and lower limbs, according to the lower-limb Vicon Plug-in-Gait marker configuration (VICON, Oxford Metrics, Oxford, UK). All children walked barefoot at a self-selected walking speed along the walkway. A trial was considered successful when there was good marker visibility. At least three successful trials were collected per participant.

Gait cycle events of initial contact and toe-off were visually determined in Nexus, Vicon (Oxford Metrics Group, UK) which was also used to apply the lower limb Plug-in-Gait for extracting 3D kinematic data. Further analysis was only carried out on the sagittal plane knee kinematics of the most affected knee organized in gait cycles. Firstly, statistical analyses of the entire kinematic waveforms were carried out in order to identify those phases of the gait cycle that significantly differed pre-post intervention. Secondly, the minimum knee extension angles during stance and during swing were calculated. These angles were previously found to be sensitive to treatment with BoNT-A in the semitendinosus (12), and to spasticity (20).

#### Instrumented Assessments During Passive Stretch

The degree and type of stretch reflex activation in the semitendinosus and its effect on passive knee extension was assessed by combining surface EMG, inertial measurement units and a hald-held dynamometer via a custom-built modular measurement system (compactRIO, National Instruments, Austin, Texas). The method has been extensively validated for use in children with CP, proving reliable and sensitive to the effects of BoNT-A (16, 21).

In children with unilateral CP, only the affected side was tested. In children with bilateral involvement, the most involved side was tested. The most involved side was defined as the side with the highest Modified Ashworth Scale score in the hamstrings (22) or, in case of symmetrical scores, an earlier knee catch angle as defined by the Modified Tardieu Scale (23). When sides were equally affected, the left side was selected. Patients lay supine and were instructed to relax. Circular Ag/AgCl electrodes (diameter of 2 cm) were placed on the muscle bellies of the semitendinosus and the rectus femoris, on specific landmarks with an inter-electrode distance of 2 cm according to the SENIAM guidelines. To minimize cable related movement artifacts on the EMG signal, EMG cables and receivers were taped to the skin. EMG data were collected using the Zerowire system (Cometa, Milan, IT) at a sample rate of 2000 Hz. EMG recording were first collected at rest to define baseline activity. With the knee and hip stabilized by the examiner at 90 degrees, children were asked to carry out three repetitions of an isometric maximum voluntary contraction (MVC) with the knee flexors. The average recorded EMG data from the semitendinosus during the MVCs were used for normalization purposes of passive stretch EMG data collected pre-treatment. Since BoNT-A influences the EMG amplitude, the MVC pre- and post-treatment are not comparable. Therefore, much care was given to standardize the location of the EMG sensors, and EMG was not normalized post-treatment and when comparing pre to post-treatment.

Following the MVCs, the knee joint was passively moved by an examiner from knee flexion to full knee extension (from here on referred to as stretch trials). Firstly, the stretch was carried out at low velocity during ± 5s, then at an intermediate, medium velocity during ± 1s, and finally at high velocity, which was performed as fast possible. Three repetitions were carried out at each velocity. Repetitions were separated by 10 s intervals in order to avoid post-activation depression of the electrophysiological response to stretch (24).

A 6th order zero-phase Butterworth bandpass filter ranging from 20 to 500 Hz was applied to the raw surface EMG signal. The EMG envelope was extracted by taking the square root after applying a low-pass 30 Hz 6th order zero-phase Butterworth filter on the squared raw signal. Joint position and joint velocity were estimated from the data collected with the inertial measurement units using a Kalman smoother (25). From this, joint range of motion (ROM) and maximum angular velocity were obtained. All angular velocity-time profiles were bell-shaped. By visualizing the raw and processed data in a custom-made Matlab GUI, stretch repetitions were excluded when performed out of plane [see Supplement 1 in (21)], at inconsistent velocities between different repetitions within a velocity trial (difference of >5, 15, or 30°/s for low, medium, and fast stretch trials, respectively), in case of poor quality EMG (loss of signal, low signal-to-noise ratio or obvious artifacts), or in case of unexpected antagonist activation.

Stretch reflex sensitivity is related to the conditions from which the stretch is performed, including starting muscle length, stretch velocity and baseline muscle tone (26). To check whether these parameters were equal across groups and sessions, EMG recordings collected at rest, the average starting knee angular positions and maximum angular velocities from slow and fast stretch trials, and the average knee joint ROM from slow stretch trials, were extracted.

To quantify the amount of muscle activation during stretch, we studied the area under the EMG envelope relative to the position moved during stretch trials:

$$\begin{array}{r} \left(1\right)integral\;EMG\;envelope=\;\int_{t_{2}}^{t_{1}}EMG_{envelope}\left(t\;\right)dt\\ \left(2\right)average\;EMG\;envelope=\frac{integral\;EMG\;evelope}{t_{2}-t_{1}} \end{array}$$

**Figure 1:**
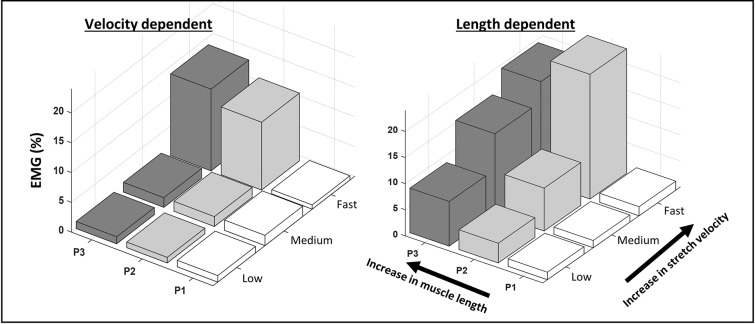
Examples of velocity- and length-dependence of the stretch reflexes in the semitendinosus. The average normalized EMG envelope collected from the semitendinosus during passive knee extensions is shown across equally spaced zones of the range of motion (P1-3) at low, medium and fast stretch velocities. In the velocity-dependent activation pattern, EMG increases with increasing stretch velocity, but not across position zones. In the length-dependent activation pattern, EMG increases with stretch velocity and across position zones.

Where *t*_1_ and *t*_2_ are times corresponding to particular joint positions. Length-dependent muscle activation, termed EMG_LD_, was expected to increase between the start and end of the slow stretch trials. Therefore, EMG_LD_ was calculated from slow stretch trials as the average EMG envelope during 60–90% ROM minus the average EMG envelope during 10–30% ROM. The extremes of the ROM (0–10 and 90–100%) were excluded as they were more likely influenced by the performance of the examiner and the comfort of the patient. EMG_LD_ was then expressed as a percentage of the pre-treatment average EMG envelope during the MVCs. Muscles with EMG_LD_ values ≥5%, were categorized as length-dependent (LD) and muscles with EMG_LD_ values <5%, as velocity-dependent (VD). Examples are provided in Figure 1.

In addition, the influence of increasing muscle stretch velocity on EMG amplitude was quantified using data from both the slow and the fast stretch trials. Specifically, the average EMG envelope during fast trials was computed over a time interval starting 200 ms prior to the time corresponding to maximum angular velocity and ending at the time corresponding to 90% of the ROM. Then, the average EMG envelope in the time interval corresponding to 10–90% ROM of the slow stretch trials was deducted from this value. This parameter is referred to as EMG_VD_, and was calculated for all muscles, irrespective of categorization as LD or VD.

After accounting for the forces caused by gravity and inertia [based on estimates of the lower leg weight (27)], the net knee torque applied by the examiner was calculated from the external forces, moments, and measured moment arms [Figure 1B in (21)]. The (compensated) knee torque at 70 degrees knee flexion (an angle that corresponded to the overall mid-ROM of all subjects), further referred to as “Torque,” was computed and the average value during slow stretch trials deducted from that during fast stretch trials. All data processing, visualization and analyses were carried out with custom-made MATLAB software (version 8.5.0 R2015a).

### Statistical Analysis

Distribution normality of the data, divided by LD and VD groups, was assessed with Shapiro-Wilik tests and by inspection of *q–q* plots. Pre-treatment data were compared between LD and VD groups, while analysis of the effect of treatment was carried out separately for LD and VD groups as well as on the entire sample. Firstly, the knee kinematic waveforms in the sagittal plane during gait were compared with two-tailed unpaired (LD vs. VD) and paired (pre-post treatment) tests using a statistical parametric (SPM{t}) or non-parametric (SnPM{t}) map, over the gait cycle data (28). The minimum knee extension angles during stance and during swing were calculated per gait cycle and averaged per individual.

Discrete parameters (from passive stretch trials and gait) pre-treatment as well as the pre-post treatment change values were compared between LD and VD groups using unpaired *t*-tests or Mann Whitney U tests. Per group, as well as for the entire group, the effect of treatment on all discrete parameters (from passive stretch and gait) were carried out using paired *t*-tests or Wilcoxon Signed rank tests. Data of the groups were then combined to explore the relations between pre-post treatment changes in passive stretch parameters with changes in discrete gait parameters using either Pearson or Spearman correlation coefficients.

For all tests, the significance was corrected for multiple testing [*p* < 0.025 according to *p*_critical_ = 1–(1–α)^1/N^ (29)]. In addition to significance, pre-post treatment change values had to be larger than the minimal detectable change values extracted from previous reliability analyses on similar samples (21, 30). All statistical analyses were carried out using SPSS (IBM Statistics 24) and custom-made scripts in MATLAB (version 8.5.0 R2015a).

## Results

### Subjects and Treatments

Included subject characteristics are reported in Table 1. Nine semitendinosus muscles were classified pre-treatment as having VD and nine as LD pattern. Children classified with VD patterns, tended to have a greater number of previous BoNT-A injections compared to those classified with LD patterns. Assessments were performed on average 59 ± 13 days after BoNT-A treatment. On average, 4.4 ± 1.0 units/kg BoNT-A were injected in the medial hamstring muscles (semitendinosus, semimembranosus and gracilis). Other muscles that received treatment are listed in Table 2. Post BoNT-A, the children wore lower and/or upper leg casts for an average of 12.67 ± 1.97 days (ranging from 10 to 14 days).

### Gait Analysis

**Figure 2:**
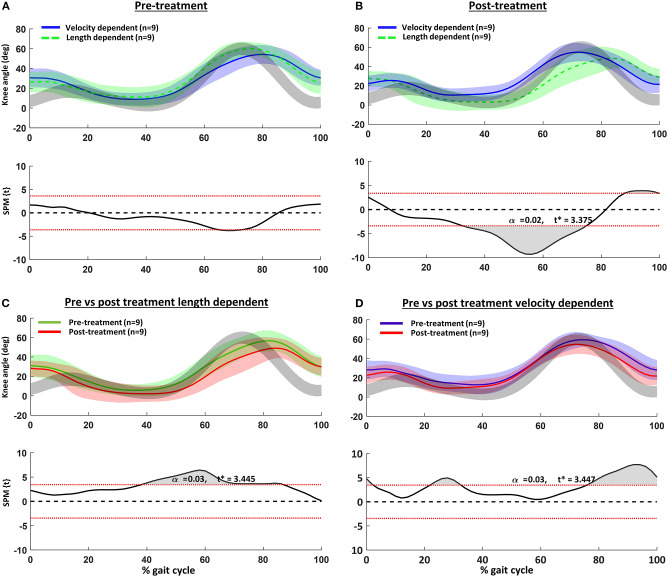
Knee kinematics in the sagittal plane vs. normalized gait cycle and results of the statistical parametric mapping (SPM): **(A)** pre- and **(B)** post-treatment velocity vs. length-dependent muscles; **(C)** pre- vs. post-treatment for the length dependent muscles; and **(D)** pre- vs. post-treatment for the velocity dependent muscles. Gray curves are from a representative database of typically developing children.

The children's knee kinematic waveforms before and after treatment together with age-related normative database of the gait laboratory (gray bands) are displayed in Figure 2. There were no significant differences in the pre-treatment knee kinematic gait curves between children with pre-treatment VD and LD muscles (Figure 2A). On the other hand, post-treatment, children categorized with an LD pattern showed significantly greater knee extension from mid- to terminal stance (Figure 2B). Those children with a pre-treatment VD pattern indicated greater post-treatment knee extension at initial contact, mid-stance and swing (Figure 2D). Comparison of the discrete gait parameters per group can be found in Table 4.

### Instrumented Assessments During Passive Stretch

EMG_LD_ pre-treatment in those muscles categorized as LD was 45% higher than those muscles categorized as VD, indicating correct group allocation. In addition, pre-treatment EMG_VD_ was about 2-fold in muscles categorized as VD compared to LD. On the other hand, the pre-treatment baseline average EMG envelope, starting angular positions, ROM, and applied stretch velocities were not different between groups (Table 3).

**Table 3:** Average (and standard deviation) values of pre-treatment parameters in velocity-dependent and length-dependent muscles from the instrumented assessments during passive stretch.

	**Pre-treatment stretch reflex activation pattern**		
**Passive stretch parameters**	**Velocity dependent (*n* = 9)**	**Length dependent (*n* = 9)**	***p*-value**
Starting position slow (deg)	114.9 (9.7)	118.2 (7.1)	0.419
End position slow (deg)	43.8 (10.5)	43.0 (8.0)	0.849
Starting position fast (deg)	115.9 (8.6)	118.9 (6.2)	0.400
End position fast (deg)	37.6 (6.8)	43.6 (9.9)	0.165
Max velocity slow (deg/s)	23.2 (3.5)	25.9 (7.3)	0.358
Max velocity fast (deg/s)	293.5 (29.8)	261.4 (33.2)	0.049
ROM slow (deg)	70.0 (6.8)	75.7 (12.6)	0.315
Torque (Nm)	8.6 (3.8)	9.1 (3.2)	0.752
Baseline EMG during slow stretch trials (%MVC)	2.6 (1.7)	7.7 (9.5)	0.065
Baseline EMG during fast stretch trials (%MVC)	2.8 (2.0)	8.4 (10.5)	0.067
EMG_LD_ (%MVC)	1.6 (1.6)	21.6 (23.8)	*0.012*
EMG_LD_ (μV)	2.2 (2.4)	15.8 (7.0)	*<0.001*
EMG_VD_ (μV)	25.7 (12.2)	11.6 (5.2)	*0.019*

*Max, maximum; ROM, range of motion; EMG, electromyography; MVC, maximum voluntary contraction; EMG_LD_, length dependent activation; EMG_VD_, velocity dependent activation. p < 0.025*.

Post-treatment results are reported in Table 4. Passive ROM significantly increased only in the VD group (+18%), while joint torque significantly decreased for the whole group (−30%) and for the LD group (−44%), but not for the VD group (−13%). EMG_LD_ significantly decreased in the whole group (−53%) and in the LD group (−68%), but tended to increase, though not significantly, post-treatment in the VD group. EMG_VD_ significantly decreased post-treatment by 60% for the whole group, and the reduction was significantly greater for the VD compared to LD group (−72% vs. −50%, *p* = 0.005). Significant positive correlations were found between the reduction in EMG_VD_ and increased knee extension at terminal swing (*r* = 0.61, *p* = 0.021).

**Table 4:** Average and standard deviation values of parameters from the instrumented assessment during passive stretch and from the knee gait kinematics in the sagittal plane during gait pre- and post-treatment.

	**All muscles (*****n*** **=** **18)**			**Velocity dependent (*****n*** **=** **9)**				**Length dependent (*****n*** **=** **9)**				**(VD vs. LD change)**
	Pre	Post	*p*-value	Pre	Post	Change	*p*-value	Pre	Post	Change	*p*-value	*p*-value
**Passive stretch parameters**												
ROM slow (deg)	72.9 (10.5)	79.2 (8.0)	0.210	70.0 (6.8)	82.7 (6.0)	12.7 (10.4)	*0.011*	75.2 (12.6)	76.4 (8.6)	1.2 (9.0)	0.683	*0.023*
Torque (Nm)	8.9 (3.5)	6.0 (3.4)	*0.003*	8.6 (3.8)	7.0 (3.1)	1.6 (2.5)	0.107	9.1 (3.2)	5.2 (3.5)	4.0 (2.7)	*0.001*	0.077
EMG_LD_ (μV)	9.8 (8.7)	4.6 (4.2)	0.366	2.2 (2.4)	4.5 (4.4)	−2.2 (4.5)	0.205	15.8 (7.0)	4.7 (4.3)	11.2 (6.5)	*<0.00*	*<0.001*
EMG_VD_ (μV)	17.9 (11.3)	6.1 (3.8)	*0.002*	25.7 (12.2)	7.3 (3.8)	18.4 (8.9)	0.012*	11.6 (5.2)	5.3 (3.8)	6.6 (5.1)	*0.003*	0.002*
**Gait analysis parameters**												
Min knee angle in stance (deg)	9.5 (8.1)	5.3 (8.3)	*0.008*	12.2 (8.9)	7.2 (7.7)	5.1 (6.5)	0.066	6.8 (6.8)	3.4 (9.0)	3.4 (5.1)	0.069	0.579
Min knee angle at terminal swing (deg)	27.7 (9.4)	24.1 (9.5)	*0.026*	27.6 (10.1)	21.1 (10.9)	6.5 (5.3)	0.012*	27.8 (9.2)	27.1 (7.5)	0.7 (5.0)	0.707	0.041

LD, length dependent; VD, velocity dependent. *Wilcoxon's signed rank test for non-normally distributed data. p < 0.025.

## Discussion

In this study, we evaluated the outcome of BoNT-A treatment in the semitendinosus in children with CP on the impairment and activity level. We tested the hypothesis that the type of pre-treatment hyper-activation of tonic stretch reflexes during passive stretch (being velocity- or length-dependent) affects the outcome.

With the exception of highly trained athletes (31), a very high stretch velocity is needed to activate a tonic stretch reflex in a healthy relaxed muscle. In contrast, in subjects with an upper motor neuron lesion diagnosed with spasticity, this threshold is markedly reduced as stretch reflexes are hyperactive (32). Clinically, this results in an exaggerated velocity-dependent resistance to passively imposed movement about a joint (33). However, studies have reported that very low velocity joint rotations (<10–35 degrees/s) may already elicit a response in subjects diagnosed with spasticity (34). This would imply that in some cases, mechanisms other than velocity-dependency are at work when a muscle involuntarily contracts in response to an externally applied stretch. Furthermore, the dependency on velocity and/or length has been described as being muscle, subject and pathology-dependent (6, 7, 35). The current study is the first to report that, at least in the semitendinosus muscles of children with CP, the presence of length-dependent activation of tonic stretch reflexes helps characterize response to focal tone-reduction treatment. Those semitendinosus muscles with more length-dependency tended to react less favorably to treatment as assessed passively and during gait.

In children with CP, spasticity in the semitendinosus is thought to restrict knee extension at terminal swing, resulting in reduced step length and excessive knee flexion at initial contact (36, 37). In accordance, pre-treatment, the knee kinematic gait pattern of the children included in the current study had excessive knee flexion in the second half of swing and during the first 10% of stance (Figure 2A). Flexion at these time points was slightly more exaggerated in those children categorized as having VD semitendinosus muscles, but the differences were not significant. Post-treatment, improvements of the knee angle at initial contact and terminal swing were only significant in those children with pre-treatment VD patterns. In contrast, post-treatment, children with LD patterns indicated knee angles approaching hyperextension in stance, as well as reduced and delayed knee flexion during swing. This pattern was also evident pre-treatment in the LD group, but was even more noticeable post-treatment.

In an extensive review of the effects of BoNT-A on gait in children with CP, Nieuwenhuys et al. (12) reported that increased knee extension in stance, similar to that observed in our LD group, was the only significant effect at the level of the knee. If our findings are confirmed, lack of reported response in other critical gait phases such as initial contact and swing in the studies included in the review, may have been due to the inclusion of children with LD, rather than VD muscles.

Furthermore, knee hyper-extension at terminal stance is not desirable and may be indicative of remaining gastrocnemius spasticity resulting in a pathological plantarflexion knee extension couple (38). While LD is less common in the gastrocnemius than in the semitendinosus (6), it may be possible that those subjects with LD semitendinosus muscles also have LD activation of their gastrocnemius and therefore reduced treatment effects. Another possibility for knee hyperextension at terminal stance is quadriceps weakness. This suggests that it may be worthwhile to explore possible differences in underlying muscle strength between LD and VD muscles. If LD muscles are indeed weaker, it may be hypothesized that the benefits of tone reduction do not outweigh the loss of muscle strength. Alternatively, LD muscles may potentially benefit from additional strength training to outbalance the loss of muscle strength caused by treatment.

Although positive treatment response as assessed during passive stretch was evident in both groups, the reduction in EMG_VD_ was significantly greater in those muscles with a pre-treatment VD categorization. This reduction was associated with an improved knee angle at terminal swing, confirming that treatment of hyperactive tonic stretch reflexes improved the children's gait pattern, in particular in those subjects categorized as having muscles with VD patterns.

Unexpectedly, muscles categorized as LD, rather than VD, demonstrated a reduction in the torque measured during passive stretch. This parameter was calculated by subtracting the torque measured during low velocity stretch from that during fast stretch at a given joint angle (70 degrees knee flexion). Therefore, a change in joint angle, rather than torque also determines the outcome of this parameter. Since ROM improved in the VD group, the lack of significant reduction in torque at 70 degrees knee flexion in the VD group may be a result of a changing joint angle-moment relationship.

Pre-treatment values of EMG_LD_ helped categorize subjects into activation patterns. Therefore, EMG_LD_ was not included in the estimation of treatment response. Furthermore, given the definition of spasticity as velocity-dependent, changes in EMG_LD_ were not deemed directly meaningful to judge the effect of BoNT-A. Nevertheless, it was expected that BoNT-A results in general muscle paresis. This was indeed confirmed, since EMG_LD_ in muscles categorized as LD decreased significantly post-treatment. It is surprising however, that in VD muscles, the opposite effect was observed, i.e., EMG_LD_ tended to increase post-treatment. Yet, this increase was not significant and values were marginal to measurement errors reported from similar samples (21).

### Limitations

The current study is not without significant limitations. Firstly, the study sample was small and heterogeneous, which did not allow us to explore more between-group differences. In particular, there were slightly more children classified as GMFCS level 1 in the VD group. Therefore, the distinction of the groups based on activation dependency alone, should be interpreted as preliminary. Additionally, previous studies included mixed forms of length and velocity dependency (6, 7). Such a mixed form is also evident in the LD example of Figure 1. Here, LD activation during slow and medium velocity stretch trials increases with increasing muscle length. Yet, this is not the case at fast velocity. This suggests an interaction between length and velocity dependent activation. While the current study is underpowered to do so, it may be interesting to further analyze the significance of these mixed patterns.

Secondly, while we refer to the semitendinosus only, its activity cannot actually be separated from that of the semimembranosus with surface EMG. Similarly, other muscles crossing the knee may have influenced the assessment of ROM and the calculation of net joint torque. Thirdly, some of the variation in post-treatment knee flexion during gait may be ascribed to the multilevel BoNT-A treatment and not by solely targeting the semitendinosus. Nevertheless, selected muscles and dosages were similar between groups (Table 2). Some subjects in the current study were also prescribed long-leg removable casts post- BoNT-A injection. While the number of children who received this were equally distributed among the groups, we are missing information regarding post-treatment cast adherence which may possibly have also influenced the results. Similarly, while all children received standardized post-treatment physiotherapy according to our hospital recommendations, we cannot exclude the possible effects of physiotherapy on treatment success.

### Possible Etiology of Length and Velocity-Dependent Activations

The etiology behind why some muscles are more length, rather than velocity-dependent is unknown and beyond the investigations of the current paper. Nevertheless, there are some possibilities that may be considered.

#### Biomechanical Orientation

During passive muscle lengthening, motor neuron activation will be defined by excitation of different receptors: muscle-velocity sensing group Ia afferents, mono- and di-synaptic group II afferents that respond to a muscle's actual length, and poly-synaptic receptors from cutaneous mechanoreceptors, joint and tendon afferents. How and which sensors respond depends on the muscles starting lengths, morphological properties, stiffness, orientation of muscle fibers with regard to its tendon, as well as spinal and supraspinal control. Moreover, these characteristics are initially defined by posture and the muscles position within the limb. For example, studies have indicated biomechanical relationships between stretch reflex activation and starting muscle lengths (26, 39, 40). In the current study however, starting posture and joint orientation were similar between subjects and can therefore not explain the observed differences in activation thresholds between LD and VD muscles.

#### Muscle Properties

Compared to typically developing children, marked morphological alterations have been reported in the semi-tendinosus of children with CP, including smaller and shorter muscle bellies, and shorter fascicles that result in an altered moment-angle relationship (41). Short, stiff muscles may activate sooner as information is relayed more efficiently between fibers (3). Evidence for this has been reported in the elbow flexors of stroke survivors and in the medial gastrocnemius muscles of children with spastic CP, where muscle activation during slow passive joint rotation was related to reduced absolute fiber length (39) and reduced muscle lengthening (42). Therefore, it is likely that altered intrinsic muscle properties in CP are partly responsible for lowered activation thresholds during passive stretch.

In many studies examining muscles affected by spasticity in the passive state, EMG activation during low-velocity passive joint rotation is regarded as indicating lack of muscle relaxation, and the data is discarded (4, 41, 43). We are fairly confident that the data presented in the current study did not reflect active participation by the subject. We were rigorous in monitoring both agonist and antagonist muscles during the passive stretch trials and carefully inspected the timing of any activation. Therefore, data were only selected from stretch trials where activation at low velocity gradually increased over the ROM. Our findings suggest that, rather than disregarding this data, capturing it may help identify children who require a different treatment approach.

#### Treatment History

There are concerns in the clinical community that repeated treatments with BoNT-A may irreversibly affect muscle volume and quality. If the hypothesis mentioned in the previous paragraph (i.e., that shorter, stiffer and more atrophied muscles elicit earlier stretch reflexes and thus LD patterns) is true, then it may be expected that those children with LD patterns would have undergone a larger number of previous treatments with BoNT-A. However, in our study, children with VD semitendinosus muscles tended to have a larger number of previous treatments with BoNT-A. Therefore, the suggestion that repeated BoNT-A detrimentally affects muscle integrity is not directly supported by our results. Rather, our finding that children with VD patterns more often underwent BoNT-A injections may reflect an appropriate subject selection and clinical decision-making. However, these are assumptions that need to be verified in future research. Given ongoing concern (14), careful monitoring of the long term effects of BoNT-A in children with CP is paramount.

#### Baseline Tone

Recently, it has been suggested that muscle spindle firing is modulated by fiber force, secondary to history-dependent features. In other words, muscles that are pre-activated (for example, when sustaining a standing posture) fire more strongly as reaction to stretch than when fully relaxed (44). In a simulation study, De Groote et al. (45) found an interaction effect between baseline tone (indicating higher intrinsic muscle force) and a reduced knee oscillation pattern when a leg with spasticity was simulated to undergo a pendulum test. Therefore, a possible explanation for a reduced activation threshold in some spastic muscles may be a higher resting muscle tone resulting in increased force detection by the spindles. Supporting this theory, in the current study, the baseline resting average EMG envelope tended to be higher in LD compared to VD muscles.

In conclusion, the clinical picture of CP is marked by heterogeneity. This emphasizes the importance of CP research focusing on disentangling and categorizing the clinical symptoms. A traditional clustering method is by the type of motor impairment, the most accepted distinguishing spastic, from dyskinetic and ataxic forms (1). Clinicians encountering mixed patterns of motor impairment, provide a diagnosis according to the most dominant and direct their treatment accordingly (1). We suggest that even within those muscles termed as spastic, deeper characterization of the reaction to passive stretch may be clinically meaningful and help direct treatment. Half of the muscles investigated in this small study sample demonstrated an activation pattern during passive stretch disparate to their original diagnosis of velocity-dependent spasticity. It is possible that these muscles possess factors that interact with their stretch reflex control. This possibly more complex disorder may explain their reduced post-treatment reaction at the muscle and joint level as well as reduced functional adaptability. If the current results are validated in larger studies, alternative treatments and closer follow-up may be more desirable than BoNT-A in children with LD muscles.

## Data Availability Statement (MS)

The raw data supporting the conclusions of this article will be made available by the authors, without undue reservation, to any qualified researcher.

## Ethics Statement

The studies involving human participants were reviewed and approved by KU Leuven University Hospitals' Ethics Committee (B32220072814). Written informed consent to participate in this study was provided by the participants' legal guardian/next of kin.

## Author Contributions

LB-O, EA, and KD conceptualized the methods. LB-O processed the data. LB-O performed the formal analysis. LB-O, KD, GM, and AV acquired funding. LB-O, KD, GM, and AV conducted the investigation. LB-O, EA, and KD developed the methodology. LB-O, KD, GM, and AV administrated the project. LB-O, KD, GM, and AV provided resources. LB-O and EA developed the software. KD and GM supervised the project. LB-O, KD, GM, and AV validated the research outputs. LB-O and EA prepared the data visualization. LB-O drafted the manuscript. All authors edited the manuscript.

## Conflict of Interest

The authors declare that the research was conducted in the absence of any commercial or financial relationships that could be construed as a potential conflict of interest.
